# A four-compartment model for the COVID-19 infection—implications on infection kinetics, control measures, and lockdown exit strategies

**DOI:** 10.1093/pcmedi/pbaa018

**Published:** 2020-05-28

**Authors:** Tianbing Wang, Yanqiu Wu, Johnson Yiu-Nam Lau, Yingqi Yu, Liyu Liu, Jing Li, Kang Zhang, Weiwei Tong, Baoguo Jiang

**Affiliations:** Peking University People's Hospital, Beijing 100044, China; Key Laboratory of Trauma and Neural Regeneration (Peking University), Ministry of Education, Beijing 100044, China; Peking University People's Hospital, Beijing 100044, China; Department of Applied Biology and Chemical Technology, Hong Kong Polytechnic University, Hong Kong, China; Gennlife (Beijing) Technology Co Ltd, Beijing, China; Gennlife (Beijing) Technology Co Ltd, Beijing, China; School of Medicine, Hangzhou Normal University, Hangzhou 311121, China; Center for Biomedicine and Innovations, Faculty of Medicine, Macau University of Science and Technology, Macau, China; Gennlife (Beijing) Technology Co Ltd, Beijing, China; Peking University People's Hospital, Beijing 100044, China; Key Laboratory of Trauma and Neural Regeneration (Peking University), Ministry of Education, Beijing 100044, China

**Keywords:** COVID-19, exit strategy, population infection rates, control measures

## Abstract

**Objective:**

To analyse the impact and repercussions of the surge in healthcare demand in response to the COVID-19 pandemic, assess the potential effectiveness of various infection/disease control measures, and make projections on the best approach to exit from the current lockdown.

**Design:**

A four-compartment model was constructed for SARS-CoV-2 infection based on the Wuhan data and validated with data collected in Italy, the UK, and the US. The model captures the effectiveness of various disease suppression measures in three modifiable factors: (a) the per capita contact rate (β) that can be lowered by means of social distancing, (b) infection probability upon contacting infectious individuals that can be lowered by wearing facemasks, personal hygiene, etc., and (c) the population of infectious individuals in contact with the susceptible population, which can be lowered by quarantine. The model was used to make projections on the best approach to exit from the current lockdown.

**Results:**

The model was applied to evaluate the epidemiological data and hospital burden in Italy, the UK, and the US. The control measures were identified as the key drivers for the observed epidemiological data through sensitivity analyses. Analysing the different lockdown exit strategies showed that a lockdown exit strategy with a combination of social separation/general facemask use may work, but this needs to be supported by intense monitoring which would allow re-introduction/tightening of the control measures if the number of new infected subjects increases again.

**Conclusions and relevance:**

Governments should act early in a swift and decisive manner for containment policies. Any lockdown exit will need to be monitored closely, with regards to the potential of lockdown reimplementation. This mathematical model provides a framework for major pandemics in the future.

## Introduction

The novel coronavirus (SARS-CoV-2) and the infection-related disease (COVID-19) were declared a public health emergency of international concern by the World Health Organization in early 2020, and have since grown into a pandemic.^1,2^ COVID-19 has created an unprecedent global health problem, for which most healthcare systems were not well prepared.^3^

Policies such as case isolation, social distancing, travel restriction, and quarantine represent the key measures adopted by various governments to control the outbreak.^4–7^ However, such measures also carry significant impact to individual psychological well-being and social/economic costs. Many epidemiological models^8–11^ have been proposed to describe the dynamics of the transmission and simulate the course of the outbreak. However, few studies have assessed the impact of the effectiveness of various measures in the control of viral spread. A four-compartment model was established to describe the SARS-CoV-2 infection, assess the potential effectiveness of various infection control measures, and make projections on the best approach to exit lockdown.

## Methods

**Figure 1.**
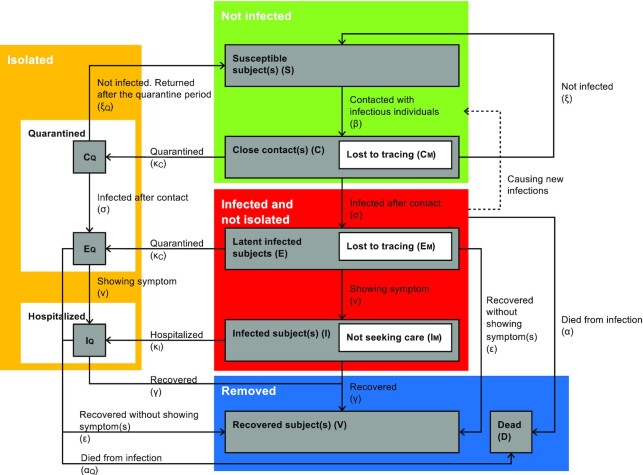
Flow diagram of the model. The four-compartment model of disease transmission incorporates the viral transmissibility and the impact of quarantine and social distancing. The population is divided into the following states: susceptible subject(s) (S), had close contact(s) (C, those that were exposed to the infected subjects/pathogen but not necessarily infected), latent (E, infected and infectious but asymptomatic), infected (I; and symptomatic), recovered (V), and dead (D). C_M_ is the portion of the contact cases that are missed by contact tracing and will not be quarantined. Individuals in states C, C_M_, and C_Q_ will progress to their respective latent groups E, E_M_ (by contact tracing), and E_Q_ (quarantined). After the onset of symptoms, latent individuals will enter the infectious status I, and I_Q_ denoting the infected population treated in isolation wards. It was assumed that when the infected subjects have recovered, they will acquire immunity that does not wane during the timeframe of the analysis (i.e. of this season).

The population is divided into the following states: susceptible subjects (S), had close contacts (C, those exposed to infected subjects/pathogen but not necessarily infected), latent (E, infected and infectious but asymptomatic), infected (I; and symptomatic), recovered (V), and dead (D) (Fig. 1 and Supplementary data).

The transmissibility of SARS-CoV-2 is modelled by two separate parameters—the social transmissibility factor β, which measures the probability of having close contact with infectious subjects, and the pathologic transmissibility σ, which measures the probability of an individual developing COVID-19 upon contact with the pathogen.^12^ The model also allows a predetermined portion of infected individuals to stay latent for the entire incubation period and then move directly to the removed states (recovered or deceased) while bypassing the infected (I) compartment.

The model was established based on demographic and COVID-19 epidemiological data in Wuhan. Data from Italy, the United Kingdom (UK), and the United States (US) fit well with our model, assuming that these countries were affected by multi-sources at around the same time. β in the community was estimated separately. All other parameters were set to the estimated parameters from Wuhan data before 23 January 2020.

## Results

### The four-compartment model and the validation

In our four-compartment Susceptible-Quarantined-Infected-Removed (SQIR) model, the transmissibility of COVID-19 is modelled by two factors, the per capita contact rate (β, social interaction factor, when multiplied by the ratio of infectious individuals in the population, describes the probability of a subject moving from status S to status C), and infection rate upon contact (σ, the viral transmission factor, the probability of a subject moving from status C to status E). Together with the quarantine rates (κ_C_ and κ_E_), they make up the parameters that can be modified by public health policies to suppress the outbreak. All other parameters in the model are pathogenic/viral characteristics that would not be affected significantly by non-pharmaceutical interventions. The progression rates from latent to infected and from infected to recovered were based on published estimates of 0.1 and 0.06^13^, respectively, which should remain relatively constant throughout the outbreak.^14–16^ The natural infection probability upon contact was set at 0.2.^12^ The rate of COVID-19-related death of all hospitalized cases was set at 4.5%.^17^

**Figure 2.**
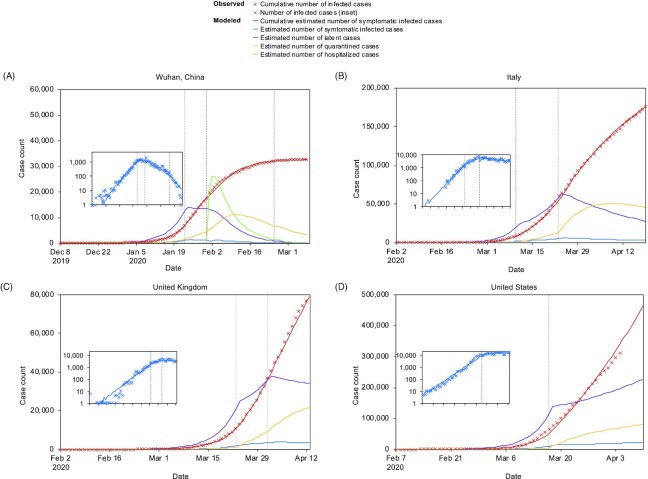
Observed and modelled case epidemic trajectories. (**A**) Wuhan, Hubei, (**B**) Italy, (**C**) UK, and (**D**) the US. Crosses represent the cumulative numbers of cases observed. Curves represent the model fitted to the observed data using MLE. Insets: the observed number of cases by date of illness onset (crosses) and the fitted curve in logarithmic scale. In Wuhan, Hubei, four distinct periods were defined: (a) before 23 January 2020 before major public health interventions, (b) between 23 January and 31 January, when there was a travel ban and cancellation of social gatherings [which would lower per capita contact rates (β)] and compulsory facemask use [which would lower the infection rate upon contact (σ)], (c) between 31 January and 17 February, when quarantine was in place, and (d) between 17 February and 18 April. In Italy, three distinct periods were defined: (a) from 26 January 2020 (5 days before the first COVID-19 confirmed in Rome) to 9 March when the nationwide lockdown was implemented; (b) from 9 March to 22 March; and (c) after 22 March when stricter lockdown policies were implemented that halted all nonessential operations. In the UK, two periods were defined before and after the nationwide lockdown was implemented on 23 March. In the US, two periods were defined before and after the ‘shelter in place’ order in the San Francisco Area on 16 March.

Our model was calibrated^18^ using 32 583 laboratory-confirmed COVID-19 cases in Wuhan, China between 8 December 2019 and 8 March 2020^19^ (Fig. 2A). The date of the first human COVID-19 latent infection (D_0_) was set as 3 December 2019 (assuming 5 days before the first symptom of patient zero). The estimation of β was 5.8 under normal social circumstance, and 1.4 after lockdown, and σ was estimated to be 0.04 in the second and third periods, representing roughly a 2-fold decrease from 0.08 during the first period possibly related to the stringent compulsory facemask use policy. Local government estimated that the effective quarantine rate after 31 January 2020 was from 35% to 75%.

### Application of the model to Italy, the UK, and the US

In Italy, our model fits, assuming there were four effective sources in Italy at around that time (Fig. 2B).^20^ The estimated β was 5.5 before social distancing (slightly lower than Wuhan, a metropolitan area densely populated). After nationwide lockdown, β was reduced to 2.8 (starting 10 March) and further reduced to 1.4 (starting 23 March), suggesting that the stricter lockdown in Italy achieved the same effect on β as in Wuhan. Our model assumed 10% effective facemask use compared to Wuhan (σ = 0.076).

For the UK, our model estimated that the number of effective sources was 6 and β was 4.6 (Fig. 2C). Nationwide lockdown was implemented on 23 March (β = 2.3, σ = 0.076) and strengthened on 31 March (β = 1.5, σ = 0.076).

For the US, based on CDC data (5 days before the first illness onset), the estimated number of effective sources was 100 (Fig. 2C). Note that the effective number of sources might come down to six, assuming the infection arrived in the US in mid-January 2020.^21^ The recent report that the SARS-CoV-2 in Washington State had the same genotype as Wuhan, whereas Northeast US had predominately the genotypes related to Europe was consistent with our projection.^22^ The estimates of β before and after the ‘shelter in place’ order were 5.2 and 2.05, respectively, indicating that US compliance was within reasonable limits and the big jump in numbers was likely related to the multiple sources of virus arriving in the US at the same time.

### Effect of case isolation and quarantine

This model projected that implementation of case isolation/quarantine is an important measure to control this pandemic. In Wuhan, combining social distancing and compulsory facemask use capped the growth rate of infected cases per day, but not enough to reverse the trend. Adding contact tracing and quarantine (and other measures including general use of facemask) 54 days after D_0_ completely curbed the outbreak in 69 days. Over the entire course, 32 583 individuals (0.3% of the population) were infected. The model projected that if no quarantine was taken and infection allowed to spread until herd immunity established, 10 111 537 individuals (91.1% of the Wuhan population) would be infected (including 4 494 017 being asymptomatic) at the end of 1 year. For comparison, the annual culminative attack rates of two common human coronaviruses 229E and OC43 were 2.8% and 26.0%, respectively.^23^

### Sensitivity analyses

To determine the sensitivity of each parameter, they were evaluated/matched to the observed outbreak data^24^ (Supplementary Table 1 and Supplementary Fig. 1). Only the control measures were found to significantly affect the outcome.

### Estimation of hospital burden

Assuming that 15%–25% of all hospitalized individuals needed critical care,^17,25,26^ the model estimated that the UK's need for hospital beds would plateau after 17 April at around 23 000 and critical care beds around 4600, close to the estimates given by NHS England.

For the US, the need for hospital beds and critical care units is still growing. Our model estimated that on 18 April 2020, the US needed 98 702 hospital beds (30 per 100 000 people) and around 20 000 critical care beds, and by 15 May (as predicted by our model on 28 April 2020), the US would need an additional 183 320 hospital beds (55 per 100 000 people) and around 37 000 critical care beds. As of 18 April, CDC reported that the overall cumulative hospitalization rate was 29.2 per 100 000 people. By the World Bank's estimation, the US currently has around 1 million hospital beds and 115 000 critical care beds, and, therefore, the COVID-19 pandemic poses a heavy burden on the US healthcare system (the need for 20% more inpatient and 35% more critical care beds). The fact that COVID-19 is very concentrated in New York City/New Jersey suggests even higher projected needs there.

### Quarantine rate and quarantine starting time

The quarantine starting time after D_0_ was identified and the effective quarantine rate had the most impact to the outcome (Supplementary Fig. 2). Sensitivity analysis of the quarantine rate of asymptomatic infected subjects (κ_E_) showed a clear breakpoint between 40% and 50%. Quarantine rates lower than 40% would lead to a completely uncontrollable outbreak.

The impact of a delayed quarantine on the effectiveness of infection control was also significant (Supplementary Fig. 2). By reducing the duration between D_0_ and the start date of quarantine measures (assuming 80% quarantine rate) from 9 to 7 weeks reduced the overall attack rate from 0.3% to 0.029%. If quarantine measures started after 11 weeks, these measures would not control the outbreak.

### Estimating the effect of lockdown and general facemask use

We evaluated the impact of lockdown by estimating the projected equivalence of lockdown/social distancing to quarantine (Supplementary Fig. 3A). A stringent lockdown reduced the average social contact by > 2-fold, equivalent to 18% effective quarantine rate. However, the breakpoint of lockdown (i.e. the β that can control the outbreak on its own) was between 0.5 and 1, much lower than the estimated β achieved by lockdown in Italy, the UK, and the US. Thus, the effectiveness of such policy in these countries was reduced as observed.

In Wuhan, compulsory facemask use reduced σ by 46%, equivalent to 19% effective quarantine rate (Supplementary Fig. 3B). The breakpoint of general facemask use was beyond 100% (around 115%), indicating that general facemask use alone would be insufficient for complete control of the outbreak . This effect was witnessed in Wuhan during the second period (23–31 January). Only after compulsory facemask use was combined with lockdown and quarantine in the third and fourth period (after 31 January) did the number of new cases show a sharp downward trend (Fig. 2A, inset).

**Figure 3.**
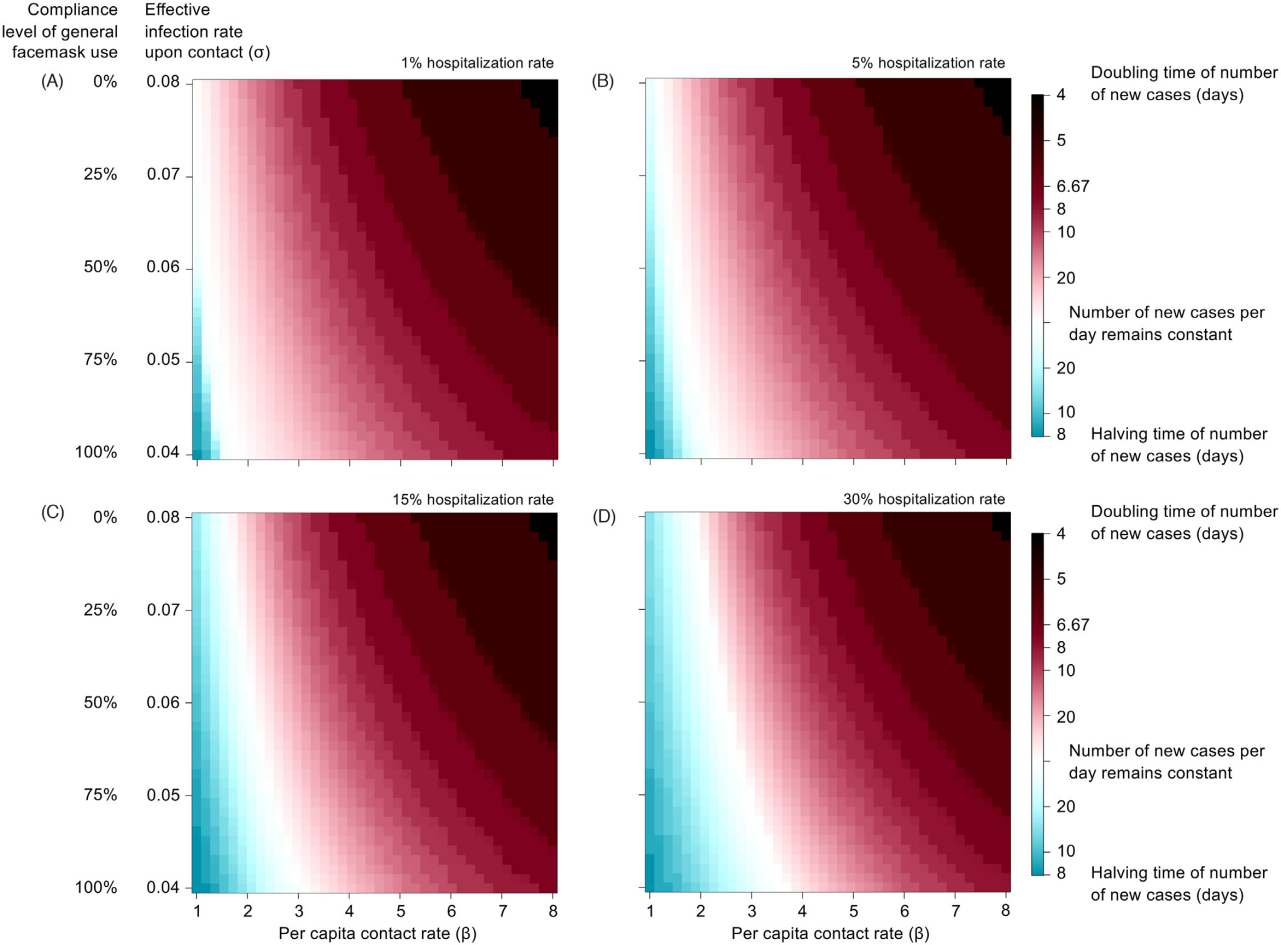
Contour plots of the effectiveness of disease control by a strategy of combining lockdown and general facemask use. The rate of disease propagation was measured by the doubling time of the number of cases, or disease suppression as measured by the time required to reduce the number of cases by half, at various compliance levels of the lockdown (as measured by the per capita contact rate) and general facemask use (as measured by the effective infection rate upon contact). (**A**) 1% hospitalization rate, (**B**) 10% hospitalization rate, (**C**) 20% hospitalization rate, and (**D**) 30% hospitalization rate. All scenarios assumed no quarantine.

The combined effect of lockdown and general facemask use given different ranges of hospitalization (1%–30%) is given in Fig. 3. During the initial surge of the COVID-19 outbreak in Italy/UK/US, no disease control policy was in place (β = 6, σ = 0.08) and hospitalization rate (κ_I_) was close to 5%, with the number of new cases per day doubling every 5 days. Both lockdown and general facemask use could reduce the growth rate of new infections; and when combined give the best overall effect. For lockdown to be effective, β needs to be reduced from 6 to < 1.1. Currently in Italy/UK/US, σ is estimated to decrease only slightly from 0.08 to 0.076. With general facemask use at 50% compliance rate (σ = 0.06), β will need to be < 1.5 to reduce the number of new infections. This level of social distancing was achieved during lockdown in Italy (estimated β = 1.44) and in the UK (estimated β = 1.5), but not yet in the US (estimated β = 2.05). With general facemask use (σ = 0.04), β will only need to be 1.99, which is slightly lower than the current US estimate (β = 2.05). If stricter lockdown and facemask use are implemented together (β = 1.5, σ = 0.04), the number of new cases would reduce by half every 16 days, even without quarantine. Higher hospitalization rate puts more cases under isolation and thus may ease the need for general facemask use. At 15% and 30% hospitalization rate, strict lockdown and compulsory facemask use (β = 1.5, σ = 0.04) would reduce new infection by half in 12 days and 10 days, respectively.

### Implications on the Italian, UK, and US control strategies and exit strategies

Currently, most government advisers recommend continuation of lockdown till the outbreak is suppressed to an acceptable level (‘Wuhan approach’), a relatively safe approach. However, some governments are considering an exit to balance the sociopsychological impact of a long lockdown and the huge impact on economy.

**Figure 4.**
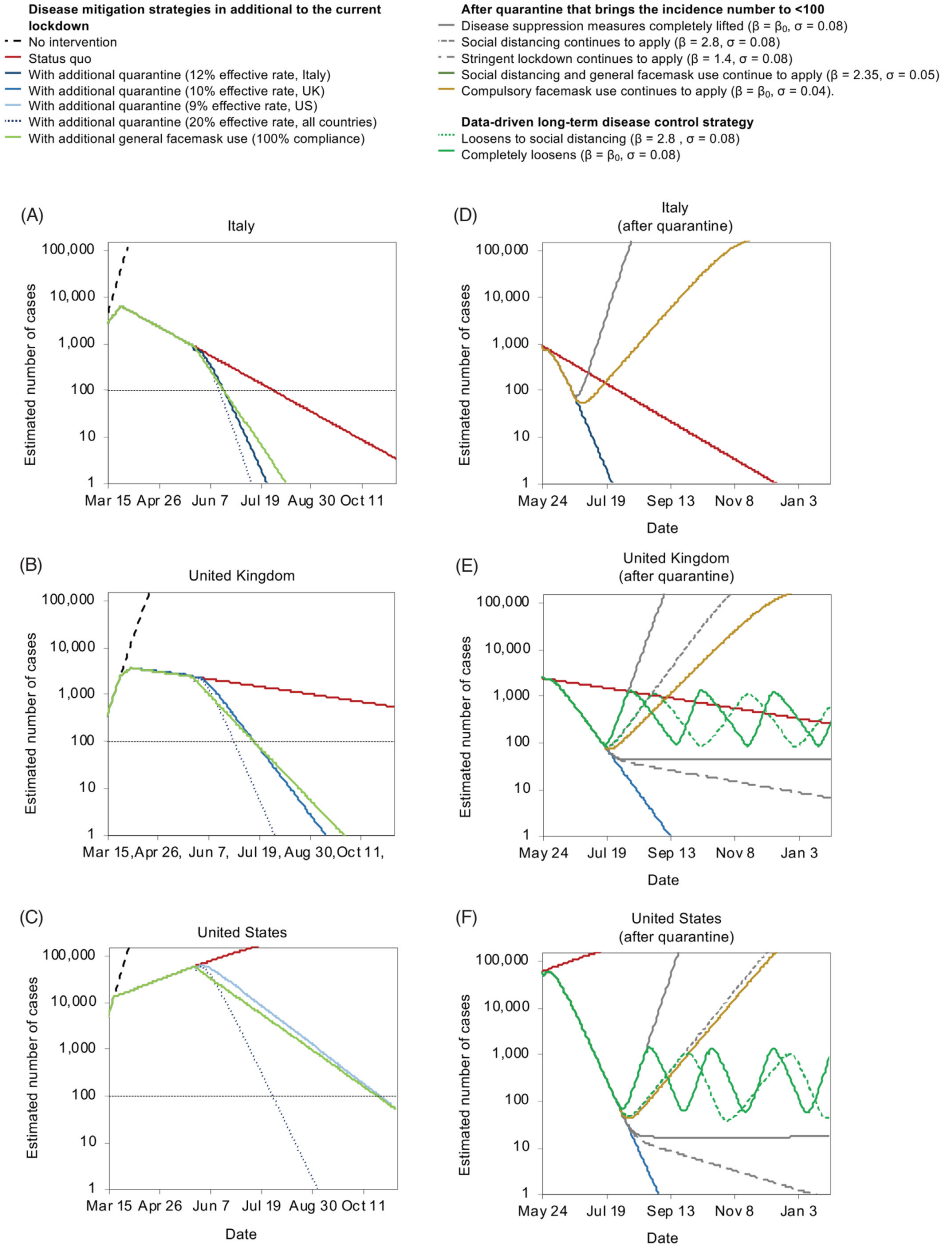
The effect of lockdown exits strategies. **(A-C)** the number of cases was compared in the following scenarios: no intervention, keeping the current public health policy, adding quarantine (at different effective ratio) to the current public health policy or adding compulsory general facemask use (at 100% compliance level) to the current public health policy: (**A**) Italy, (**B**) UK, and (**C**) US. Alternative exit strategies are compared with the current public health policy assuming quarantine to be lifted after the number of cases is below 100 in (**D**) Italy, (**E**) UK, and (**F**) US. Various level of social distancing and facemask use and data-driven lockdown policies are considered, including: disease suppression measures completely lifted (β = β_0_, σ = 0.08), social distancing continues to apply (β = 2.8, σ = 0.08), stringent lockdown continues to apply (β = 1.4, σ = 0.08), social distancing and general facemask use continues to apply (β = 2.35, σ = 0.05), compulsory facemask use continues to apply (β = β_0_, σ = 0.04). Data-driven lockdowns are also plotted: loosening of social distancing (β = 2.8, σ = 0.08) and completely loosened (β = β_0_, σ = 0.08). β_0_: per capita contact (β) rate of the normal social distance in each country. This model can be modified to adapt to different adjusted scenarios.

In Italy, for lockdown to continue till zero new infection, it would have to continue until 6 January 2021 (Fig. 4A). For the UK, it would not be until 2023 before the number of new infections dropped to zero (Fig. 4B). The US is still not seeing a firm plateau in the number of new cases and thus it may be even longer until the projection of zero new infection (Fig. 4C).

A more balanced approach would be to reduce the number of new infections to a considerably low level, then relax the infection control policies measures in a controlled fashion with intensive monitoring. Based on our projection, either strict quarantine of contacts, or a combination of both a relatively strict lockdown and general facemask use may be sufficient. It is important to note that the latter approach would need to be used in combination because of (a) the non-linear nature of the effectiveness, and (b) the existence of breakpoints as described above.

We predict that in Italy, a quarantine policy with an effective quarantine rate of 12% starting on 24 May (18 weeks after D_0_) in addition to the current implemented lockdown would reduce the incidence of new infections exponentially, reaching ≤ 100 in just 26 days (19 June) and zero in 62 days (24 July). The same infection suppression effect can also be achieved with no active quarantine, but by using the same lockdown as at present, and mandate compulsory facemask use (Fig. 4A). In the UK, lockdown and general facemask use starting on 24 May would bring the incidence of new infections to under 100 in 52 days (15 July) and to zero in 129 days (30 September) (Fig. 4B). This is almost equivalent to an effective quarantine rate of 10%. An effective quarantine rate of 20% would bring the incidence of new infections to under 100 in 35 days (28 June) and to zero in 70 days (2 August). In the US, general facemask use starting on 24 May would bring the incidence of new infections to under 100 in 152 days, which is equivalent to a quarantine effective rate of 9%. If the government can achieve 20% effective quarantine rate, the incidence of new infections will drop to around 100 in 65 days (28 July).

Implementing a monitoring-based, data-driven lockdown exit strategy is essential to sustain the containment of COVID-19. Based on our model, loosening the quarantine too early while there are still a significant number of latent cases may lead to an uncontrollable second outbreak. With daily active monitoring of new infection numbers, it is possible to adjust the infection control policies to maintain new infections within a band trending downwards. As discussed, lockdown should only be loosened to social distancing (β = 2.8) and general facemask use when daily new infections are reduced to a very low number (e.g. 100 cases) and re-implemented to an aggressive lockdown (β = 1.4, similar to the lockdown in Italy) with general facemask use when daily new infections is rising (e.g. ≥1000 cases). This approach will provide the time for vaccine, drugs, or other pharmaceutical interventions to catch up while allowing economical activities to be less uninterrupted in regions with low numbers of new infections.

For Italy (Fig. 4D), if the quarantine is lifted after daily new infections drops to < 100 (June 19) with all infection suppression policies currently in place (β = 5.53, σ = 0.08), the number of new infections would quickly return to exponential growth. Even with general facemask use (β = 5.53, σ = 0.04), a second wave of outbreak is still inevitable. Thus, the quarantine should last till the incidence of new infections is close to zero (on 24 July), plus a 2-week wash out period (the estimated latent period) before implementing exit.

In the UK, if quarantine is lifted after daily new infections drops to < 100 (15 July) along with all current infection suppression policies (β = 4.6, σ = 0.08), it would only take 36 days (20 August) for the incidence of new infections to reach 10 000. However, an extended lockdown and quarantine that lasts 129 days (until 30 September to eliminate new incidences) would also be less plausible economically and politically. Thus, we recommend that after the outbreak is suppressed to an acceptable level, restrictions can be relaxed gradually to keep the infection under control, allowing economic activities to recover.

In the US, to effectively reduce the size of the outbreak, quarantine should remain in effect at > 20% rate. With quarantine, the number of daily new cases is projected to be < 100 by 28 July. Afterwards, a monitoring-based, data-driven approach can be implemented. The same level of lockdown or social distancing plus general facemask use should continue. It should be noted that a full cycle of loosening and re-implementation would span 53 days if during the loosening period, the infection control policies were completely lifted (β = 5.2), or 89 days if loosened to social distancing (β = 2.8).

## Discussion

In this study a four-compartment mathematical model was established for the SARS-CoV-2 infection, which could be useful in the policy decision-making process. Second, our model suggested that Italy, the UK, and USA likely had multiple sources of infections to account for the observed early sharp rise in the number of infected subjects. Third, effective and early implementation of quarantine were the two most important factors for control of the outbreak. Fourth, the relative contributions of quarantine, lockdown, social distancing, and the general facemask use were estimated. Finally, different strategies for lockdown exit were evaluated and challenges identified. Our model allows examination of the issues unique to SARS-CoV-2 infection, the highly infectious nature of this pathogen, the potential of this infection to overwhelm the healthcare system, and the alternative containment strategy implemented for this pandemic.

The sensitivity analyses showed that, in the time from the index patient to control measures and effective quarantine, measures were most effective when the majority of the infected carriers, mostly asymptomatic^27–31^, were put under quarantine. Our data support the latest recommendation from the US CDC on the use of facemasks in public during this pandemic.

Once the outbreak is under control, quarantine can be lifted through a data-driven, monitoring-based dynamic disease mitigation policy. Thus, general availability of a rapid viral diagnostic test is critical. A real challenge will be the potential to have both SARS-CoV-2 and other upper respiratory viral pathogens prevalent at the same time (e.g. this winter) when this pandemic is still ongoing. If this occurs, our model will require adjustment for two pathogens simultaneously. Our data highlight the importance for governments to act swiftly and decisively for any containment policies.^32^ Also, any lockdown exit must be monitored closely with the potential for lockdown reimplementation.

## Conclusions

This four-compartment mathematical model describes SARS-COV-2 infection, can be adjusted to reflect local transmission characteristics and public health capabilities, can help to determine the optimal local disease suppression strategy, and can help when making projections for the best local lockdown exit strategy.

## Supplementary Material

pbaa018_Supplemental_File
