# Supplementary material for: A four-compartment model for the COVID-19 infection—implications on infection kinetics, control measures, and lockdown exit strategies
Source: Precis Clin Med. 2020 May 28;3(2):104–12. doi: 10.1093/pcmedi/pbaa018 (PMC7313816; doi:10.1093/pcmedi/pbaa018)
Supplement: pbaa018_Supplemental_File [file pbaa018_supplemental_file.docx]

**Supplementary Data**

**Supplementary Figures**

**Supplementary Table**

**Parameters in the Four-Compartment Model**

**Supplementary Figure Legends:**

**Supplementary Figure. 1 Sensitivity analysis.** The cumulative number of infected cases in the US as predicted by the mathematical model under various range of the parameters. **(A)** per capita contact rate (β), **(B)** infection rate upon contact (σ), **(C)** progression rate from contact to latent, **(D)** progression rate from latent directly to recovered, **(E)** progression rate from latent to infected, **(F)** progression rate from contact back to susceptible, **(G)** hospitalization rate and **(H)** quarantine rate. The dash lines indicate the parameter values calibrated to the US data.

**Supplementary Figure. 2. The cumulative number of infected cases in the US as predicted by the model.**  The situation in the US was analyzed under different disease suppression at varying start time. **(A)** lockdown (which affects the per capita contact rate), **(B)** general facemask use (which affects the infection rate upon contact, σ), **(C)** hospitalization rate and **(D)** quarantine rate.

**Supplementary Figure. 3. Equivalent quarantine rate.** The equivalence of different compliance levels of **(A)** lockdown (which affects the per capita contact rate, β) and **(B)** general facemask use (which affects the infection rate upon contact, σ) compared to quarantine.

**Supplemental Figures**

**Supplementary Figure 1.**

**
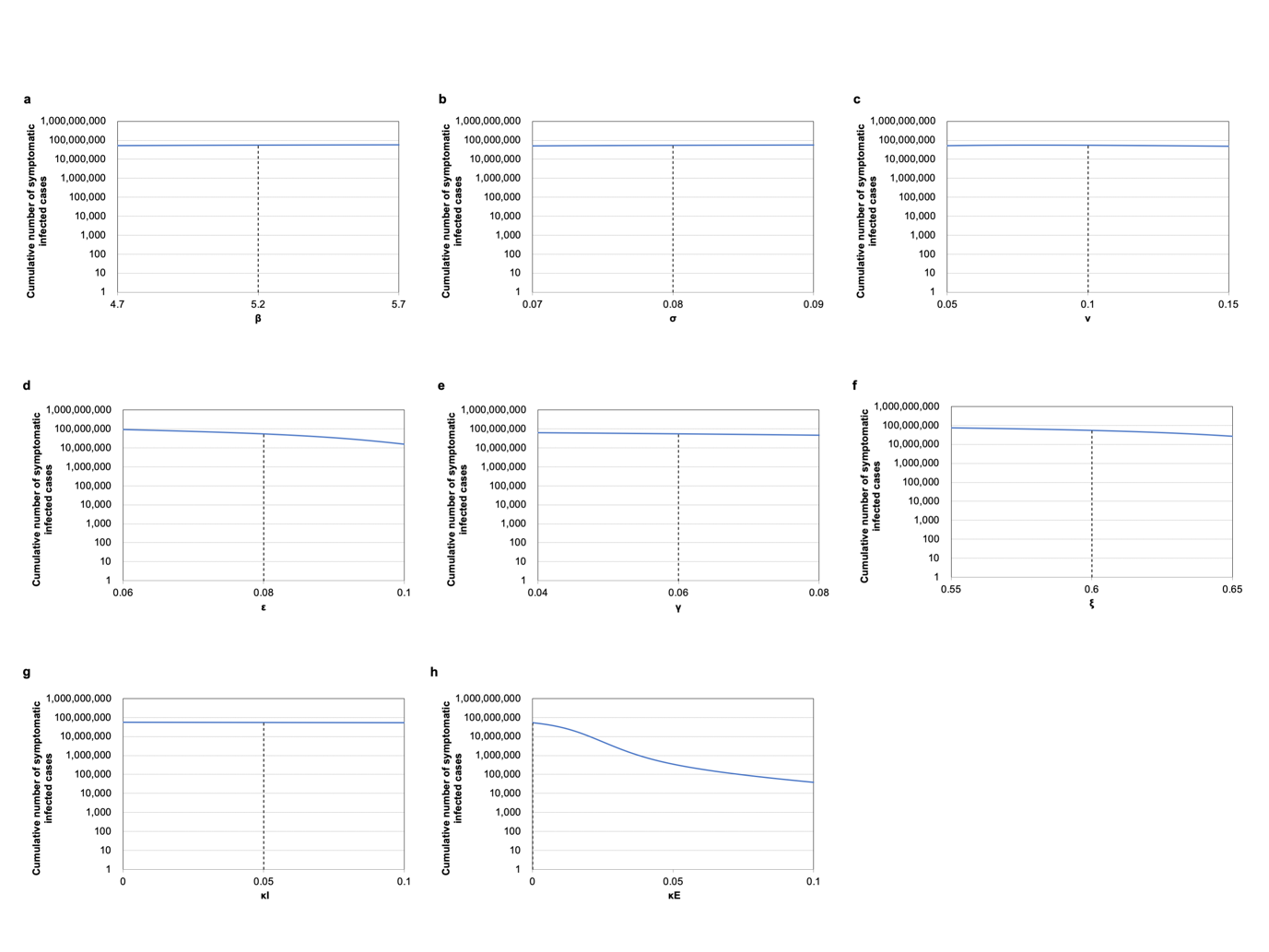
**

**Supplementary Figure 2.**


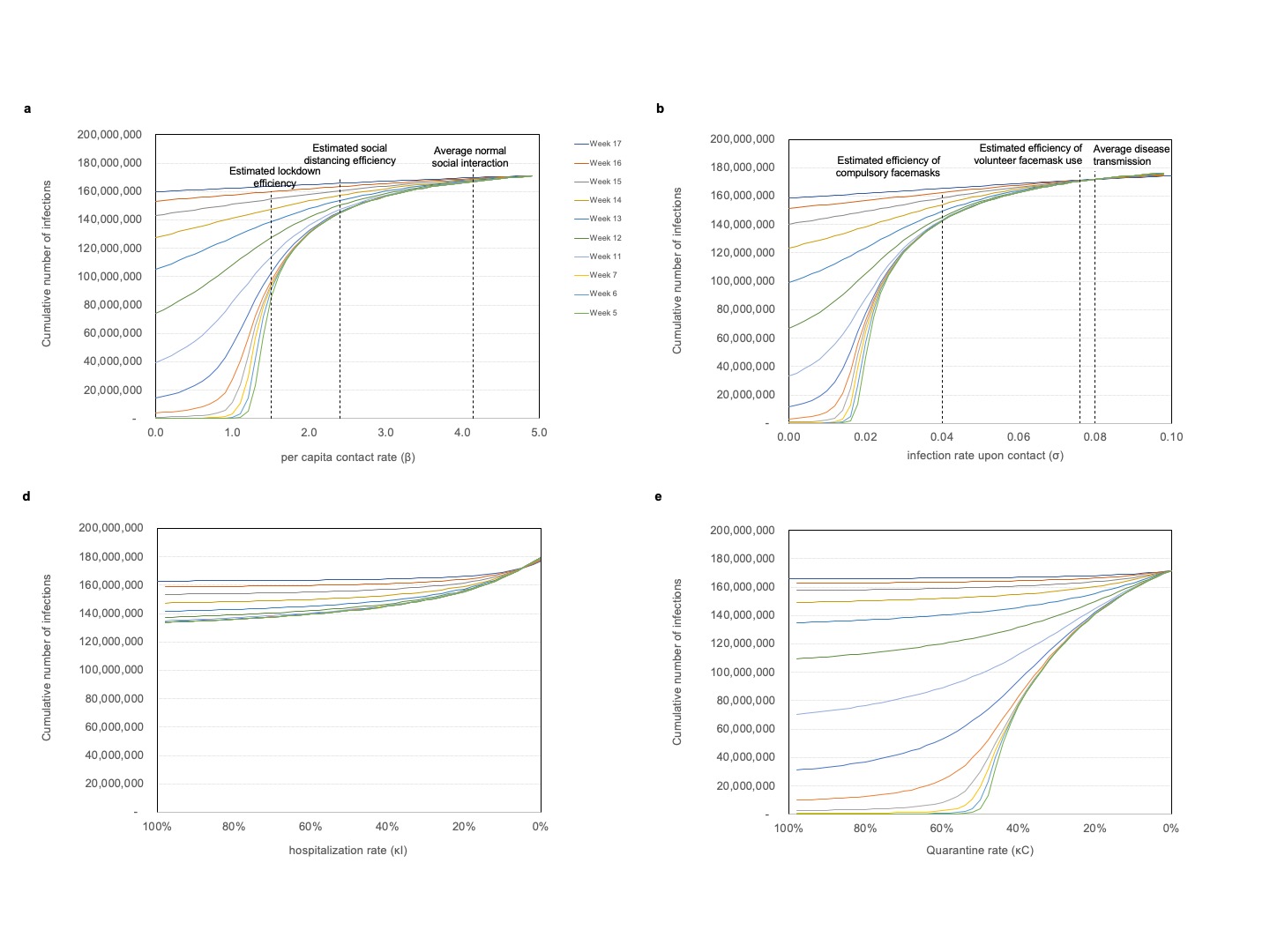


**Supplementary Figure 3.**


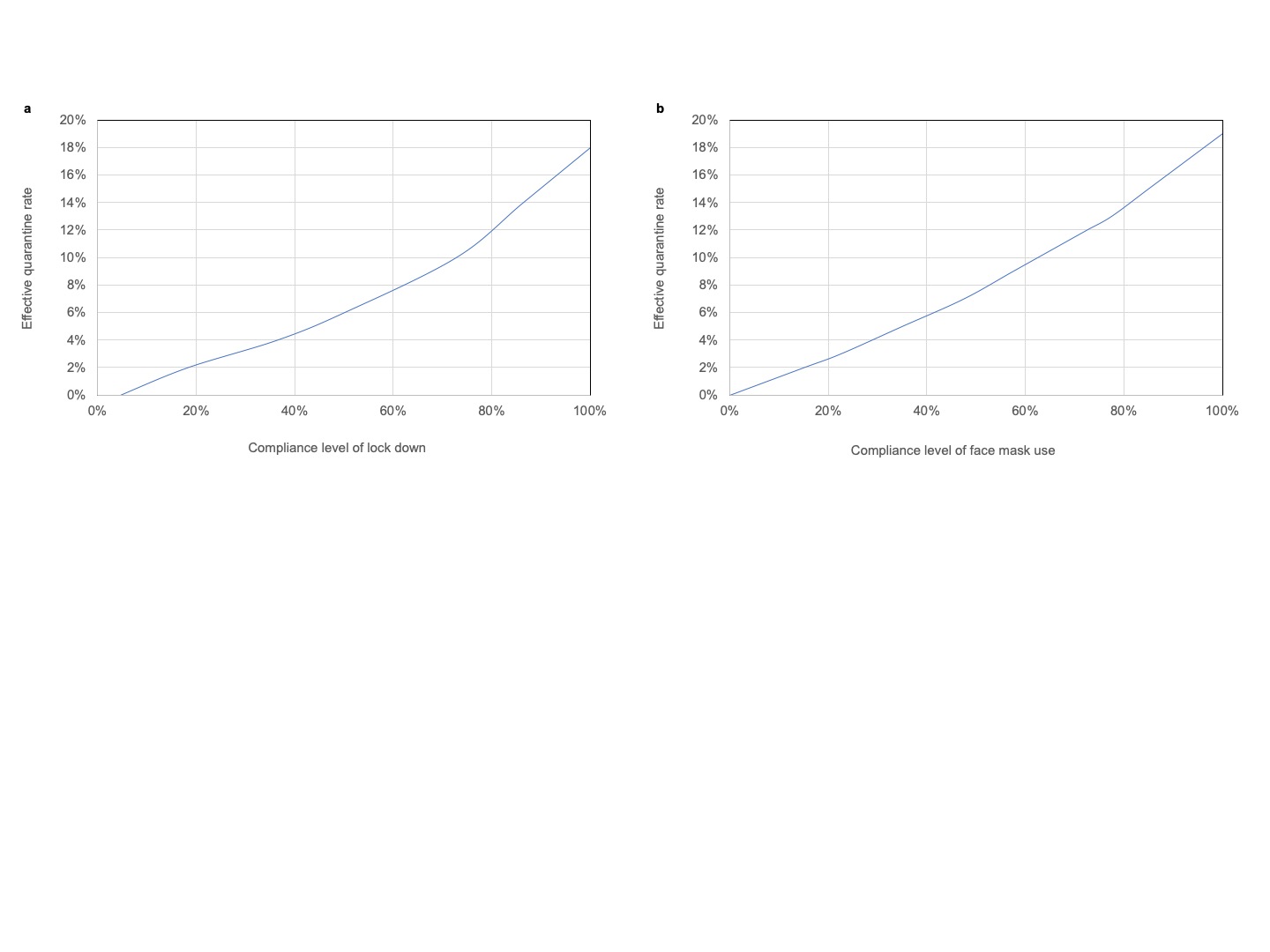


**Supplemental Table**

**Supplementary Table 1** Definition, the range of baseline value, and the sensitivity analysis of the parameters in this Mathematical Model.

| **Parameter** | **Description** | **Baseline value** | **Δ% cumulative number of cases / Δ% parameter** |
| --- | --- | --- | --- |
| β | Average per capita contact rate | 4.6 - 5.8 | 0.47 |
| σ | Infection rate upon contact | 0.2 | 0.41 |
| ξ | progression rate from contact back to susceptible | 0.6 | -0.15 |
| ν | progression rate from contact to latent | 0.1 | -2.89 |
| γ | progression rate from latent to infected | 0.06 | -0.46 |
| ε | progression rate from latent directly to recovered | 12.5 | -5.19 |
| κ_I_ | Hospitalization rate of infected individuals | 15% - 30% | -0.02 |
| κ_E_, κ_C_ | Quarantine rate of the latent individuals, individuals had close contact | 0% | -0.04 |
| S | Population in Wuhan in 2020 — permanent residents | 11.10 million |  |
| S | Population in Italy in 2020 | 60.48 million |  |
| S | Population in the United Kingdom in 2020 | 66.57 million |  |
| S | Population in the United States of America in 2020 | 331.0 million |  |
| α_Q_ | disease-induced mortality rates of all hospitalized cases | 0.045 |  |

**Parameters in the Four-Compartment Model**

The population is divided into the following states: susceptible subjects (S), had close contacts (C, those that were exposed to the infected subjects/pathogen but not necessarily infected), latent (E, infected and infectious but asymptomatic), infected (I; and symptomatic), recovered (V) and dead (D) (Figure 1). C_M_ is the portion of the contact cases that are missed by contact tracing and will not be quarantined. Individuals in states C, C_M_ and C_Q_ will progress to their respective latent groups E, E_M_ (by contact tracing) and E_Q_ (quarantined). The per capita quarantine and isolation rates are described by κ_C_ (close contact cases), κ_E_ (infected cases during latent period) and κ_I_ (infected and symptomatic cases).

After the onset of symptoms, latent individuals will enter the infectious status I, and I_Q_ denoting the infected population treated in isolation wards. We assume that when the infected subjects have recovered, they will acquire immunity that does not wane during the timeframe of the analysis (or this season).

The transmissibility of SARS-CoV-2 is modelled by two separate parameters – the social transmissibility β which measures the probability of having close contact with infectious subjects, and the pathologic transmissibility σ which measures the probability of an individual developing the COVID-19 upon contact with the pathogen^15^. Once contact cases under quarantine are determined as not infected, they are returned to the susceptible subject group at a rate ξ. The per-capita rates of progression from latent to infectious and then to recovery are denoted with the symbols ν and γ, which are inversely proportional to the mean latent period and the mean infectious period, respectively. The transition rates σ, ν and γ were assumed to be the same for the traced, missed, and quarantined subgroups, as the disease behaviour should not be affected by patient’s isolation status. The disease-induced mortality rates of all hospitalized cases (severe cases) and all other cases (mild cases) are denoted by α_Q_ and α, respectively.

The model allows a predetermined portion of infected individuals to stay in the latent state for the entire incubation period and then move directly to the removed states (recovered or deceased) while bypassing the infected I compartment^13,26,27^. The ratio of cases that remained asymptomatic until recovered (ε/ν) were set to 60% of all infections (previous reports estimated this to be between 42% to 86%)^28-31^.

The seeding time was set 5 days (i.e. average the incubation period) before the first human COVID-19 infection was reported. The model was first calibrated with the Wuhan data (assuming all infections came from a single source) using Markov Chain Monte Carlo methods with Gibbs sampling and a non-informative flat prior to the COVID-19 daily incidence data using maximum likelihood estimation approach^32^.

Datasets from Italy, UK, and the US were employed to evaluate the effectiveness of the various infection suppression strategies.
